# A nutrient relay sustains subtropical ocean productivity

**DOI:** 10.1073/pnas.2206504119

**Published:** 2022-10-03

**Authors:** Mukund Gupta, Richard G. Williams, Jonathan M. Lauderdale, Oliver Jahn, Christopher Hill, Stephanie Dutkiewicz, Michael J. Follows

**Affiliations:** ^a^Department of Earth, Atmospheric and Planetary Sciences, Massachusetts Institute of Technology, Cambridge, MA 02139;; ^b^Division of Geological and Planetary Sciences, California Institute of Technology, Pasadena, CA 91125;; ^c^Department of Earth, Ocean and Ecological Sciences, School of Environmental Sciences, University of Liverpool, Liverpool L69 3GP, United Kingdom;; ^d^Center for Global Change Science, Massachusetts Institute of Technology, Cambridge, MA 02139

**Keywords:** subtropical gyres, biological production, nutrient supply, mesoscale eddy transport

## Abstract

The expansive gyres of the subtropical ocean account for a significant fraction of global organic carbon export from the upper ocean. In the gyre interior, vertical mixing and the heaving of nutrient-rich waters into the euphotic layer sustain local productivity, in turn depleting the layers below. However, the nutrient pathways by which these subeuphotic layers are themselves replenished remain unclear. Using a global, eddy-permitting simulation of ocean physics and biogeochemistry, we quantify nutrient resupply mechanisms along and across density surfaces, including the contribution of eddy-scale motions that are challenging to observe. We find that mesoscale eddies (10 to 100 km) flux nutrients from the shallow flanks of the gyre into the recirculating interior, through time-varying motions along density surfaces. The subeuphotic layers are ultimately replenished in approximately equal contributions by this mesoscale eddy transport and the remineralization of sinking particles. The mesoscale eddy resupply is most important in the lower thermocline for the whole subtropical region but is dominant at all depths within the gyre interior. Subtropical gyre productivity may therefore be sustained by a nutrient relay, where the lateral transport resupplies nutrients to the thermocline and allows vertical exchanges to maintain surface biological production and carbon export.

## Significance

The vast subtropical oceans play a leading role in the global storage of organic carbon into the deep ocean. There, biological production is limited by the availability of surface nutrients due to the large-scale ocean circulation pushing nutrient-rich waters at depth. The transfer of nutrients into the sunlit layer is achieved by fine-scale vertical motions, at the expense of the layers beneath. We show that subsurface layers are substantially replenished by the lateral turbulent transport of nutrients along density surfaces, on 10 to 100 km scales. This nutrient relay, involving both vertical and lateral transport, ultimately fuels biological production and sustains an associated sequestration of carbon in the subtropics.

**Fig. 1.**
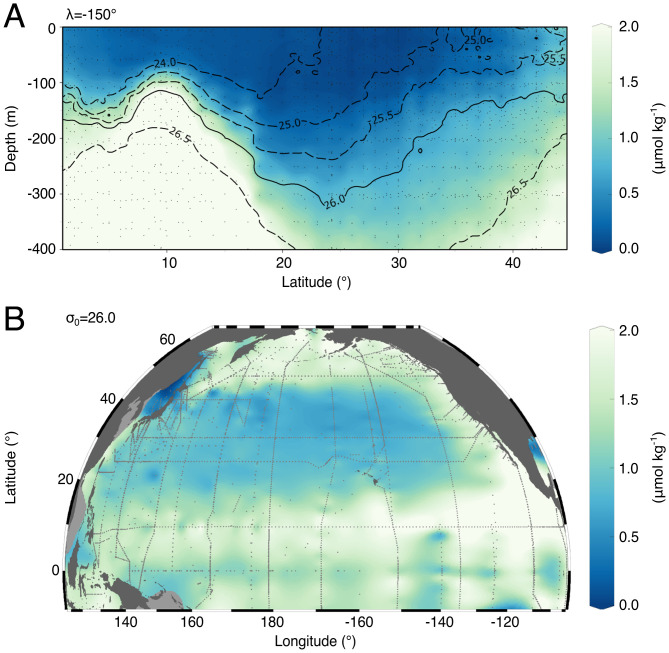
PO${}_{4}^{3-}$ concentration (μmol kg^−1^) in the North Pacific basin obtained from the Global Ocean Data Analysis Project (GLODAP) V2.2 atlas and plotted using Data-Interpolating Variational Analysis (DIVA) gridding in Ocean Data View. (*A*) Transect along 155 ^∘^W, with contours of constant density *σ_o_* (thick dashed lines) and sampling locations (gray points). The solid black line indicates the *σ_o_* = 26.0 surface. (*B*) PO${}_{4}^{3-}$ distribution along *σ_o_* = 26.0 with sampling locations indicated by gray points.

The sinking of particulate organic carbon from the sunlit euphotic zone into the deeper, dark ocean maintains an oceanic reservoir of dissolved inorganic carbon, changes in which can significantly modify atmospheric CO_2_ (1). The ocean’s subtropical gyres exhibit low surface concentrations of nutrients and biomass but, due to their very large surface area, may contribute a significant fraction of global export. For example, the North Pacific subtropical gyre is estimated to represent ~20 to 50% of the total North Pacific organic sinking flux (2, 3). The wind forcing over the subtropical basins leads to a downward doming of the density surfaces that contain an extensive volume of low-nutrient waters (4), as revealed in an observed transect from the North Pacific (Fig. 1) (5). The mode of nutrient resupply and the long-term maintenance of biological productivity in subtropical gyres have presented a conundrum for several decades. Inorganic nutrients are incorporated into photosynthetic phytoplankton and pass through the food web, but despite efficient recycling within the sunlit euphotic zone (6, 7), gravitational sinking and the subduction of organic matter deplete the surface nutrients of the subtropical gyres. These surface nutrient losses are largely viewed as being offset by the physical transport of nutrient-enriched, deeper waters back into the sunlit zone, together with smaller contributions from atmospheric deposition and nitrogen fixation. However, the nature of the nutrient pathways recharging the subtropical nutrient reservoir below the euphotic zone remains poorly constrained.

The sharp vertical gradient of nutrients just below the euphotic layer can sustain the vertical supply of nutrients up to the surface. Transfer of nutrients upward into the subtropical euphotic zone has been attributed to vertical diapycnal mixing (8–11); the passage of mesoscale eddies, which adiabatically lift nutrient-rich, subeuphotic layers into the light (12–19); and submesoscale (1 to 10 km) features that are associated with strong vertical circulations (20–24). However, all of these localized processes deliver nutrients into the euphotic layer while depleting the subeuphotic layers below (17, 25). A long-standing question is how the nutrient inventory of this subeuphotic layer, which fuels the local vertical supplies, is maintained over the longer term (26, 27).

At the flanks of the subtropical gyre, wind-driven upwelling brings nutrient-rich waters toward the surface, forming nutrient gradients in the subpolar and equatorial regions, as seen in observed transects (Fig. 1*A*). Within the frictional boundary layer of the ocean, the wind-driven, meridional Ekman transport acts on these lateral gradients to transfer nutrients into the subtropical gyre, significantly supplementing vertical processes in sustaining local productivity (4, 28, 29). However, this lateral transport contribution is confined to the upper few tens of meters and diminishes away from the gyre margins due to biological consumption (4).

**Fig. 2.**
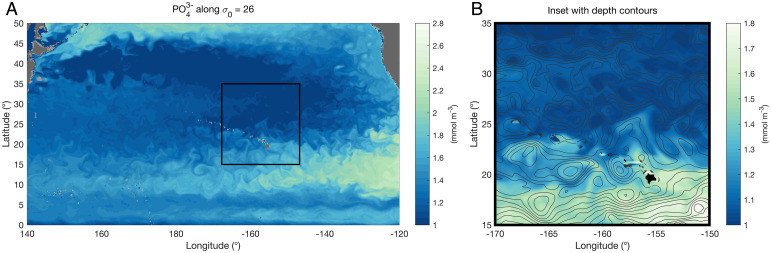
Snapshot of simulated PO${}_{4}^{3-}$ concentration (mmol m^−3^) during the month of September and along the *σ*_0_ = 26.0 isopycnal (*A*) over the north subtropical Pacific basin and (*B*) in the proximity of the Hawaiian islands. The black line contours in *B* depict the depth of the isopycnal surface with 10-m contour intervals. Mesoscale features transport nutrients into the North Pacific subtropical gyre through the combined action of eddy stirring that draws out filaments of tracer and advection by coherent eddy structures.

Mesoscale eddies can provide a lateral transport of nutrients, which involves both stirring and advective transfer along density surfaces (11, 17, 25, 30, 31). Idealized simulations and theory suggest an important combined nutrient supply to the gyre from lateral eddy diffusion and Ekman transport (30). Diagnostics of a more realistic, global eddy-resolving model show that lateral eddy transfers do provide an important nutrient flux across the boundaries of the subtropical gyre (31). Stimulated by these model-based inferences, an observational field study, measuring microscale turbulence and nutrients, reveal signals of eddy stirring along density surfaces, providing a weak nutrient supply within the thermocline over the center of the North Atlantic subtropical gyre (11). A closure for the nutrient supply then suggests that the nutrient delivery by mesoscale eddy stirring should be one to two orders of magnitude larger over the flanks of the subtropical gyre due to an increase in nutrient gradients and a greater tilt of the density surfaces (11). These enhanced nutrient gradients and isopycnal slopes are evident near the margins of the subtropical gyre, particularly at its southern flank, between *σ*_0_ = 24.0 and 26.5 (Fig. 1). An eddying numerical simulation reveals the associated mesoscale flux of nutrients, visible as streamers of high-phosphate waters emanating from nutrient-rich currents all around the margins of the subtropical gyre (Fig. 2*A* and Movie S1).

In this study, we quantify the nutrient pathways and fluxes of the subtropical North Pacific Ocean, in the context of a global eddy-permitting numerical model with explicit representation of biogeochemical and ecological processes (*Materials and Methods*). In doing so, we test and illustrate the hypothesis of ref. 11 that the lateral eddy transfer contribution is stronger at the gyre margins and in the thermocline. Complementing previous work (31), we resolve along- and across-density surface fluxes and the depth and intragyre structures in fluxes and balances, as well as the contribution of dissolved organics.

We find that a nutrient relay occurs in the subtropical gyre (11), involving the eddying transfer of nutrients from the upwelling flanks of the gyre, downward along sloping isopycnals into the subtropical gyre interior. This nutrient supply into the thermocline offsets the export of organic matter to the deep ocean and fuels upward vertical transfer to the surface, helping to sustain biological production in the euphotic layer. We illustrate this nutrient relay in the following, detailed analysis of the nutrient budget for density layers over the North Pacific subtropical gyre.

## Quantifying Contributions to the Nutrient Relay

In the ocean interior, away from the energetic influences of wind and buoyancy forcings near the surface, ocean currents flow mostly adiabatically along density surfaces (32). Carefully accounting for the transient tracer transport by the circulation is facilitated by analyzing their motion in an isopycnal coordinate system, rather than the depth level coordinates in which the model is formulated. In order to quantify the effect of mesoscale eddies, we developed an algorithm to achieve this coordinate transformation in a manner that conserves mass, such that budgets are closed exactly (details in *SI Appendix*). In isopycnal coordinates, the tracer balance can be written as follows (11, 33, 34):

[1] $$\underset{\mathrm{Tendency}}{\underset{︸}{\frac{\partial (hP_{\sigma })}{\partial t}}}\;+\;\underset{\overset{\text{Isopycnal}\;\text{flux}}{\text{divergence}}}{\underset{︸}{\nabla_{\sigma }\cdot (F_{\sigma }h)}}\;+\;\underset{\overset{\text{Diapycnal}\;\text{flux}}{\text{divergence}}}{\underset{︸}{h\frac{\partial F_{d}}{\partial \hat{d}}}}=\underset{\overset{\text{Biological}}{\text{source}}}{\underset{︸}{hB_{\sigma }}},$$

where *h* is the isopycnal layer thickness, $P_{\sigma }$ is the nutrient concentration, $B_{\sigma }$ is the biogeochemical transformation term, $F_{\sigma }$ is the nutrient flux along isopycnals, and *F_d_* is the nutrient flux across isopycnals. Both $F_{\sigma }=(F_{\sigma }^{x},F_{\sigma }^{y})$ and *F_d_* are composed of the sum of an explicitly resolved advective component and a parameterized diffusive component.

**Fig. 3.**
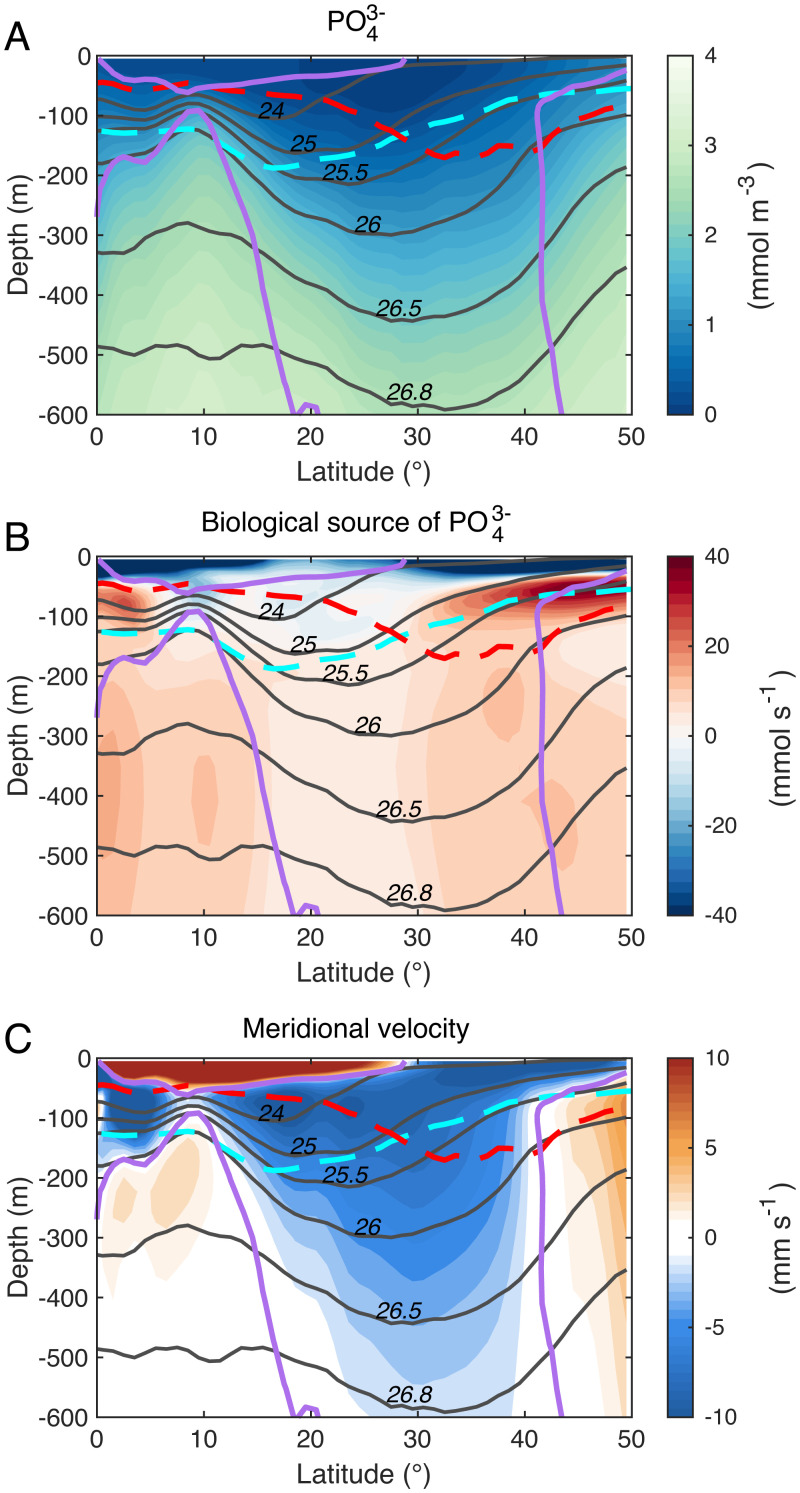
Zonal mean profiles averaged between years 1994 and 2003 over the entire North Pacific basin for (*A*) PO${}_{4}^{3-}$ (mmol m^−3^), (*B*) the volume-integrated biological source of PO${}_{4}^{3-}$ (mmol s^−1^) defined positive when PO${}_{4}^{3-}$ is supplied and negative when PO${}_{4}^{3-}$ is consumed, and (*C*) the meridional velocity (mm s^−1^) averaged over the eastern Pacific (100 ^∘^W to 180 ^∘^W) to avoid the influence of the Kuroshio current. Colored lines indicate the winter mixed layer depth (red dotted), the summer euphotic depth defined at 3 μE m^−2^ s^−1^ (cyan dotted), and contours of zero meridional velocity (purple). The gray lines depict isopycnal levels referenced at the surface.

In steady state, Eq. **1** describes how physical processes, involving isopycnal and diapycnal flux convergences, balance the biogeochemical source term shown in Fig. 3*B*. For the 10-y time period considered in this study, the tendency term is not always negligible, particularly over the strongly time-varying eddy flows of the Kuroshio western boundary current. Decadal variability likely also contributes to nutrient tendencies at the basin scale. Nevertheless, averaged over the Pacific subtropical gyre, physical processes act to replenish the euphotic layer by supplying nutrients from reservoirs at depth (*SI Appendix*, Fig. S2).

The mesoscale eddies present in the simulation transport nutrients along isopycnal surfaces with a magnitude that can be quantified by performing a Reynolds decomposition of Eq. **1** (details in *SI Appendix*). The total time mean and layer-integrated isopycnal flux $\bar{F_{\sigma }h}$ is composed of a mean component ${\left(F_{\sigma }h\right)}^{\mathrm{mean}}$ and a time-varying eddy component ${\left(F_{\sigma }h\right)}^{\mathrm{eddy}}$, such that

[2] $$\bar{F_{\sigma }h}={\left(F_{\sigma }h\right)}^{\mathrm{mean}}+{\left(F_{\sigma }h\right)}^{\mathrm{eddy}},$$

where the mean component is defined as

[3] $${\left(F_{\sigma }h\right)}^{\mathrm{mean}}\equiv \bar{u_{\sigma }}\bar{P_{\sigma }}\bar{h}$$

and depends upon the product of the time means of the velocity, nutrient concentration, and layer thickness at a fixed position. $u_{\sigma }$ is the flow velocity along isopycnal surfaces, and the overbar represents a time averaging over the simulation period. The eddying component ${\left(F_{\sigma }h\right)}^{\mathrm{eddy}}$ is obtained as a residual from $\bar{F_{\sigma }h}$ and captures the effects of correlations between the time-varying fluctuating isopycnal layer thickness h′, the along-isopycnal flow velocity $u_{\sigma }\prime$, and the nutrient concentration $P_{\sigma }\prime$. ${\left(F_{\sigma }h\right)}^{\mathrm{eddy}}$ includes both an eddy advective bolus flux and an eddy diffusive flux directed along density surfaces (35, 36), although the parameterized diffusive contributions along isopynals are less significant than their resolved advective counterpart.

**Fig. 4.**
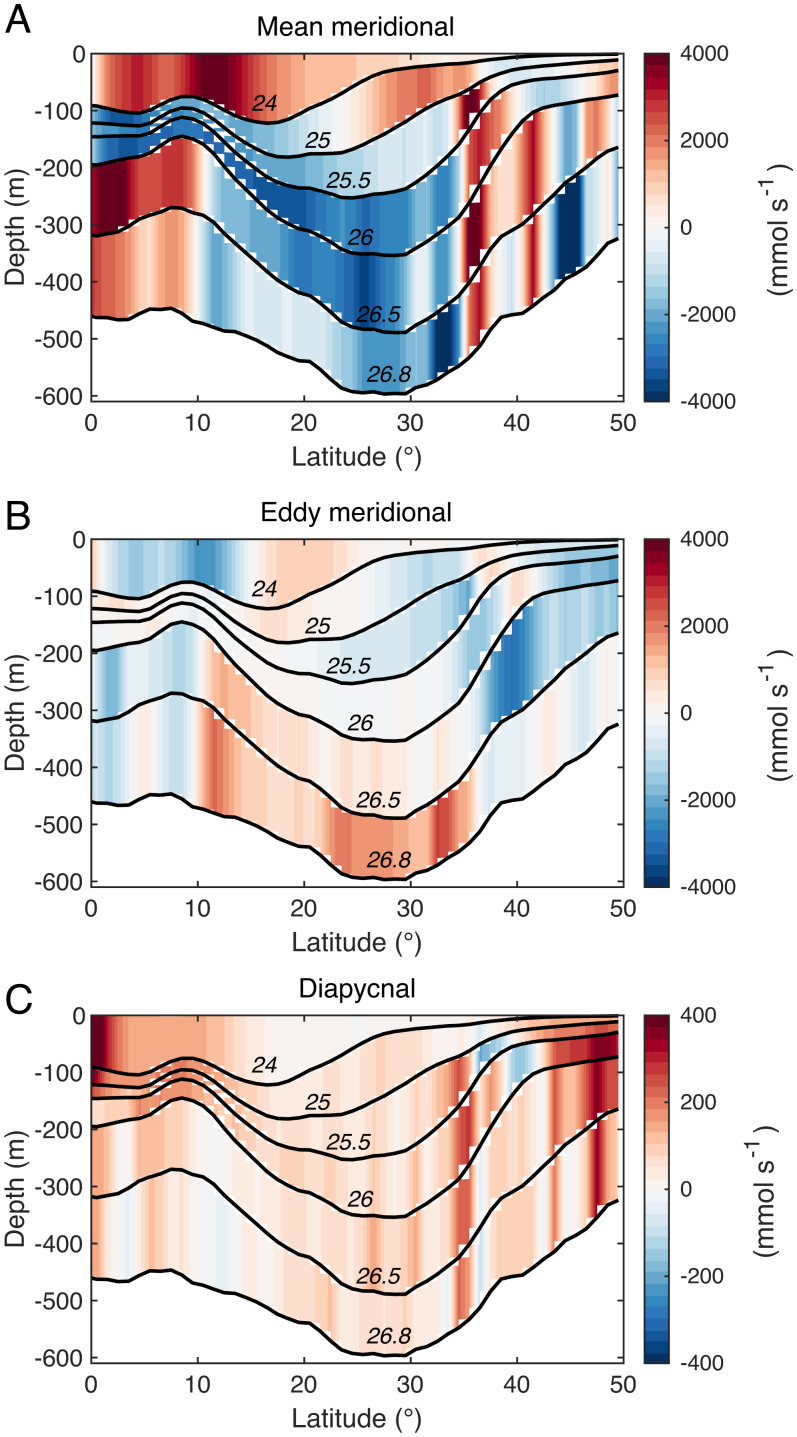
Basin-averaged and area-integrated fluxes of PO${}_{4}^{3-}$ (mmol s^−1^) calculated over six isopycnal layers ranging from the surface to *σ*_0_ = 26.8 and plotted in depth coordinates. (*A*) Meridional isopycnal mean (positive northward), (*B*) meridional isopycnal eddy, and (*C*) diapycnal flux (positive upward) evaluated using the decomposition illustrated in *SI Appendix*, Fig. S1. Note that while the magnitude of diapycnal fluxes is significantly weaker than that of along-isopycnal fluxes, their convergences are of comparable strengths (see Fig. 5).

The pattern of the basin-averaged mean flux ${\left(F_{\sigma }h\right)}^{\mathrm{mean}}$ (Fig. 4*A*) closely matches that of the time mean meridional velocity averaged over the eastern part of the subtropical Pacific, outside of the Kuroshio’s influence (Fig. 3*C*), with northward Ekman flow at the surface and southward geostrophic flow within the gyre interior, as predicted by Sverdrup theory. The eddying component of the flux, ${\left(F_{\sigma }h\right)}^{\mathrm{eddy}}$, tends to oppose the contribution by the time mean flow, particularly within the deeper part of the gyre (Fig. 4*B*), where eddies flux nutrients northward and toward the center of the gyre. At the northern flank of the subtropical gyre, the eddy flux is generally southward, which also tends to oppose the local time mean flow. Diapycnal processes mix deep, nutrient-rich waters with shallower, nutrient-depleted waters, leading to an upward (positive) diapycnal flux over most of the domain (Fig. 4*C*).

The simulated mesoscale eddies temporarily heave nutrient-rich isopycnals toward the surface, leading to a rectified nutrient source to the euphotic layer of the subtropical gyre (12). In our diagnostic framework, the eddying biological source term is calculated as part of the Reynolds decomposition (*SI Appendix*), and its effect is illustrated in *SI Appendix*, Fig. S5. Consistent with previous studies (15, 26), fluctuating isopycnal heaving provides a source of nutrients into the euphotic zone of the subtropical gyre and assists diapycnal proceses in maintaining biological productivity in the region. This effect is most significant at the margins of the gyre, and particularly over its southern flank, where nutrient-rich isopycnals are closer to the surface than in the central part of the basin. Over the subpolar gyre, this term is a net sink due to strong convective mixing in winter, which subducts surface nutrients down into the interior of the ocean (15).

## Regimes of Physical Nutrient Balance

**Fig. 5.**
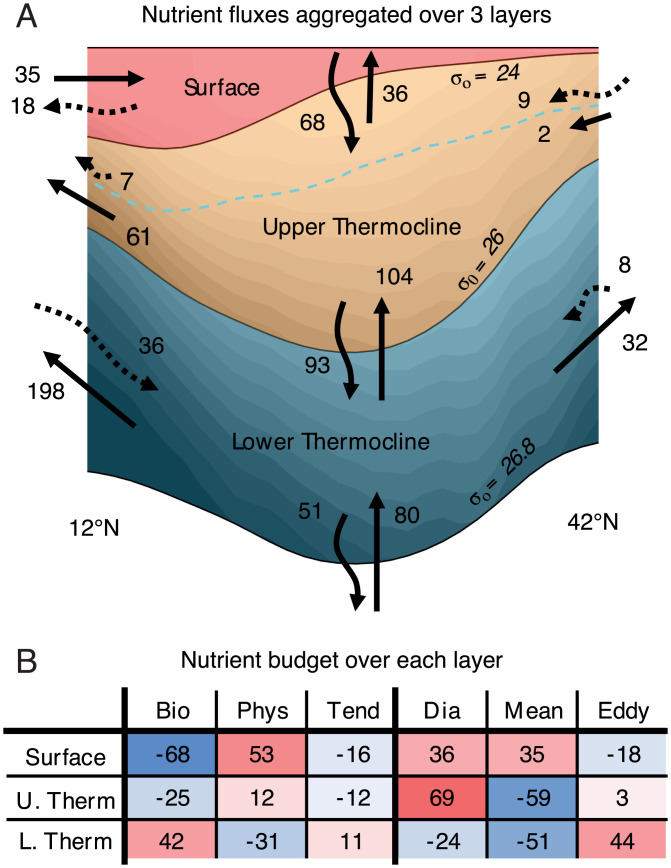
PO${}_{4}^{3-}$ budget averaged over latitudinal bands (12 ^∘^N to 42 ^∘^N) and aggregated over three isopycnal layers representative of the surface, upper thermocline, and lower thermocline layers. All quantities are in units of 10^5^ mmol s^−1^. (*A*) Schematic diagram displaying the fluxes at the boundaries of the subtropical basin. The shading represents the zonal mean PO${}_{4}^{3-}$ distribution, with darker colors within each layer depicting regions of higher concentrations. The arrows at the southern and northern edges of the diagram represent along-isopycnal time mean (full lines) and time-varying eddy (dotted lines) fluxes. The straight vertical arrows represent diapycnal transport across each layer. The wavy vertical arrows represent implied biogeochemical fluxes obtained by integrating the biogeochemical source term $\bar{hB_{\sigma }}$ over each density layer with a no-flux boundary condition at the surface. At the deeper interfaces, this implied flux is largely composed of sinking organic particles. The blue dashed line indicates the euphotic depth, as defined in Fig. 3. (*B*) Table summary of the nutrient budget over the three aggregated layers. The first three columns show the overall nutrient budget, highlighting the balance between the biological source, physical source (sum of isopycnal and diapycnal convergences), and tendency terms. The last three columns are a breakdown of the physical source term, which includes the diapycnal convergence and isopycnal time mean and time-varying eddy convergences.

Integrating the nutrient budget over the northern subtropical Pacific provides insight into the large-scale balance of physical and biological processes that shape the nutrient cycle of the region. The latitudinal bounds of the North Pacific subtropical basin are chosen as 12 ^∘^N and 42 ^∘^N, where the magnitude of the mean meridional flow away from the western boundary current approaches zero (Fig. 3*C*). The integral is performed from coast to coast, such that the net zonal flux vanishes, and only the diapycnal fluxes and the meridional fluxes along isopycnals remain in the calculation (*SI Appendix*, Eq. **S7**). Vertically, the budget is aggregated into three characteristic layers, namely, surface, upper, and lower thermocline, which display distinctive physical sources of nutrient balance and are bounded by the following isopycnal surfaces: $\sigma_{0}=$ 24.0, 26.0, and 26.8 (Fig. 5).

The surface layer (*z* = 0 to $\sigma_{0}=$ 24.0) contains the prominent northward Ekman flow at the southern flank of the gyre (Fig. 3*C*). This layer represents a large biological sink of nutrients due to uptake by phytoplankton, which is resupplied in equal measure by diapycnal fluxes from the layer underneath, and through the time mean northward Ekman flow carrying nutrients upwelled within the North Equatorial Current. In this upper ocean layer, lateral eddy transfer at the mesoscale opposes the effect of the time mean flow and diverges nutrients away from the region, consistent with the results in ref. 30. Mesoscale isopycnal heaving also supplies nutrients to the subtropical euphotic zone, a contribution which is included within the net biological source term shown in Fig. 5*B* and in *SI Appendix*, Fig. S5.

The upper thermocline ($\sigma_{0}=$ 24.0 to 26.0) is located below the ocean’s surface frictional boundary layer, in a region where the time mean flow advects nutrients away from the gyre, particularly at its southern flank. Biological production, enhanced by isopycnal eddy heaving, drives a net loss of nutrients from this layer, the upper part of which is within the euphotic zone. The nutrient sink is almost exclusively resupplied by diapycnal flux convergence, while the net effect of lateral eddy transfers is a negligible part in its overall nutrient balance.

The lower thermocline ($\sigma_{0}=$ 26.0 to 26.8), which lies entirely beneath the euphotic layer, loses nutrients to the waters above by diapycnal mixing. The time mean flow also transports nutrients away from the region, with the southward mean flow pushing against the nutrient gradient. In this dark layer, remineralization accounts for approximately half of the nutrient resupply, but a large fraction of the particulate sinks even deeper. The budget is closed by the convergence of isopycnal eddy fluxes, transporting nutrients down-gradient toward the center of the gyre at both flanks. Mesoscale eddies therefore play a critical role in replenishing the nutrients held in the lower thermocline of the North Pacific subtropical gyre which, in turn, recharges the euphotic layer through a variety of localized, vertical processes. Without this key mesoscale contribution, the nutrient inventory and productivity of the upper subtropical ocean could be significantly reduced.

The transport of dissolved organic phosphorus (DOP) also provides a source of phosphorus to the subtropical gyres, previously estimated to make a modest contribution to export in the North Atlantic (37, 38). Here simulated DOP is qualitatively consistent with observations (*SI Appendix*, Fig. S6), with highest concentrations near the surface and in the productive upwelling regimes. The simulated flux of DOP is significant in the surface layer, with a convergent supply into the gyre that is comparable to that of phosphate. In the thermocline, however, the DOP transport is an order of magnitude smaller than the phosphate flux (*SI Appendix*, Fig. S6) and does not contribute significantly to the recharging of the thermocline reservoir.

## Mesoscale Influence in the Recirculating Gyre Interior

**Fig. 6.**
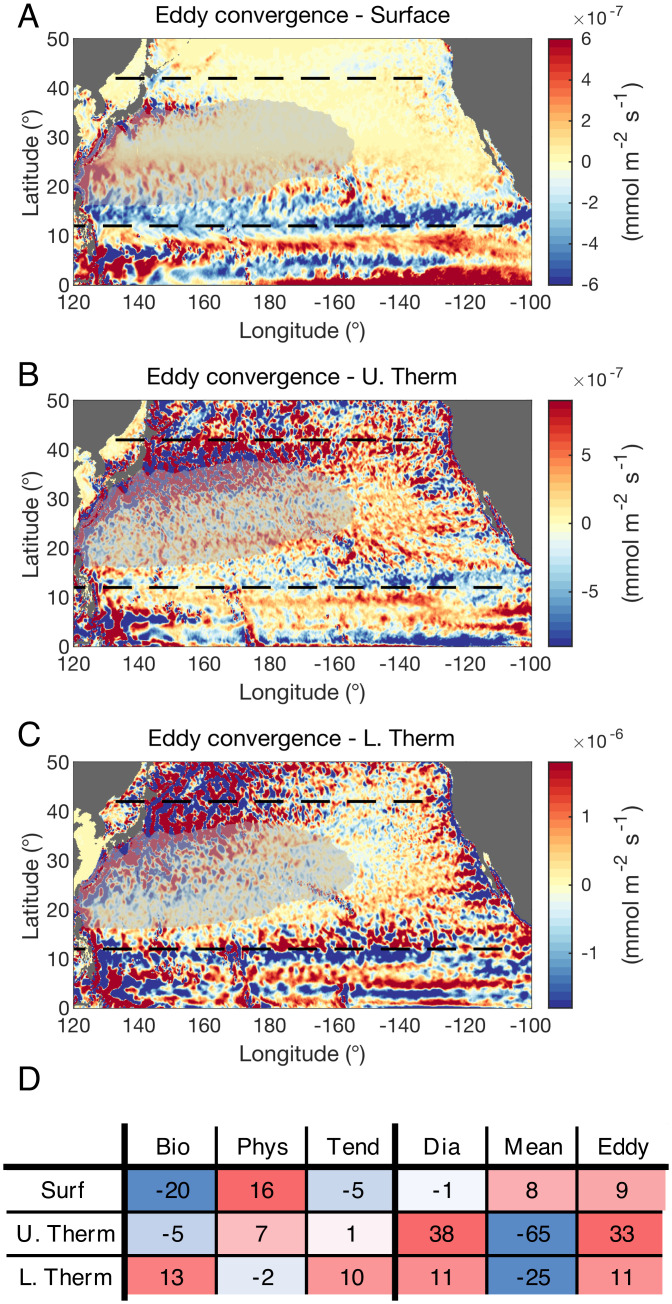
Convergences of the isopycnal PO${}_{4}^{3-}$ eddy flux (mmol m^−2^ s^−1^) over the (*A*) surface, (*B*) upper thermocline, and (*C*) lower thermocline layers. All fields are smoothed with a 2D Gaussian kernel that has an SD spanning one grid point. Different color bar ranges are employed to account for larger convergence magnitudes in deeper layers. The dotted lines at 12 ^∘^N and 42 ^∘^N delimit the meridional extent of the gyre assumed for the budget in Fig. 5. The gray transparent shading depicts the gyre recirculation interior, defined as the region where the depth of the $\sigma_{0}=26.8$ isopycnal is larger than *d_g_* = 450 m (*SI Appendix*, Fig. S4). (*D*) Tabulated summary of the nutrient budget contributions (10^5^ mmol s^−1^) over the three aggregated layers (as in Fig. 5*B*) integrated over the gyre recirculation interior. Here the eddy convergences are positive over all three layers.

While the basin mean nutrient budget highlights the overall effect of mesoscale eddies in fluxing nutrients isopycnally into the lower thermocline, it is also instructive to consider the nutrient budget in the central part of the subtropical ocean (31). In this region, the thermocline and nutrient-rich surfaces are deepest, and the upwelling regions at the flanks of the gyre are remote. We define the recirculating gyre interior as the region within the subtropical Pacific where the depth of the $\sigma_{0}=26.8$ isopycnal exceeds $d_{g}=$ 450 m, as depicted in Fig. 6. This gyre interior region is directly analogous to the western thermocline recirculation of ref. 39 and the unventilated western pool of ref. 40, where mesoscale eddies are assumed to transfer properties from a bounding streamline down gradient, leading to extensive regions of nearly uniform potential vorticity within the thermocline (41, 42). Here isopycnal eddy transports may also be particularly important for the nutrient budget of the upper and lower thermocline.

In the surface layer, mesoscale eddies transfer nutrients from the North Equatorial Current (10 ^∘^N to 20 ^∘^N) to the gyre interior recirculation. In the upper thermocline, the eddy flux convergence is visible over all the flanks of gyre recirculation region, with contributions from the North Equatorial Current in the south, the Kuroshio extension in the northwest, the North Pacific Current in the north, and the California Current to the east (Fig. 6). In the lower thermocline, the eddy contributions to nutrient convergence in the southern and eastern parts of the gyre recirculation are weaker than in the upper thermocline, but the Kuroshio extension still provides a net positive eddy flux of nutrients to the region.

When integrating over the recirculating gyre interior, all three layers reveal a significant source of eddy convergence in the nutrient budget, with a particularly large eddy contribution in the upper thermocline layer (Fig. 6*D*). As with the subtropical basin-averaged budget shown in Fig. 5*B*, the eddy convergence opposes the divergence from the time mean flow within both thermocline layers. In the lower thermocline, the diapycnal convergence diagnosed over the gyre interior differs from the diapycnal divergence that was recorded over the whole subtropical basin (Fig. 5*B*). This difference arises due to the nutrients in the thermocline being held deeper within the gyre recirculation region, which leads to the upward diapycnal transfer of nutrients occurring deeper in the water column than over the flanks of the gyre (*SI Appendix*, Fig. S4).

The salient importance of eddy convergence is robust to alternative definitions of the gyre recirculation region. Using a deeper threshold value for the isopycnal depth *d_g_* highlights the eddy transport within the upper thermocline, while a shallower value of *d_g_* provides estimates that are similar to the overall subtropical budget in Fig. 5*B*, where the eddies are most influential in the lower thermocline. We conclude that mesoscale eddies recharge the nutrient reservoir within the entire water column of the recirculating gyre interior, against the effects of the divergent time mean flow below the surface. This process helps explain the biological activity of the central part of the gyre, which accounts for ~40% of the modeled primary production within the subtropical basin, despite being a region largely depleted in nutrients in the time mean state (*SI Appendix*, Fig. S3 and Fig. 3*A*).

## Discussion and Conclusions

The maintenance of photosynthetic activity in oligotrophic subtropical gyres has been a long-standing conundrum. Large-scale downwelling induced by the wind-driven circulation, coupled with gravitational sinking of organic particles, depletes the nutrient inventory of surface waters in these subtropical gyres. A variety of localized processes deliver nutrients vertically from the upper thermocline into the euphotic layer at the expense of the nutrients held below but leave the question of how these deeper layers are subsequently recharged.

**Fig. 7.**
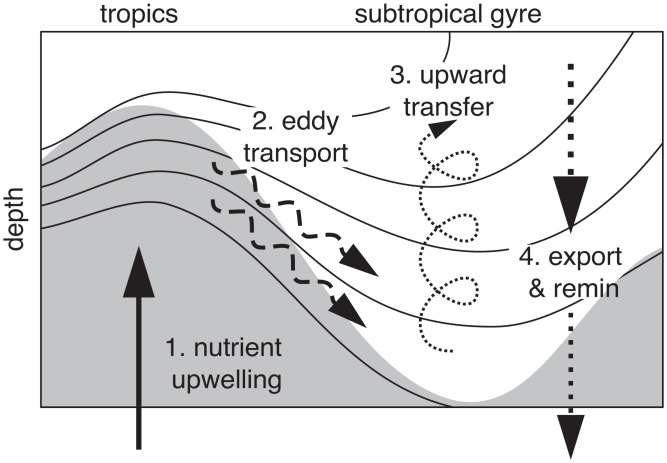
Schematic representation of the nutrient relay mechanism sustaining productivity in subtropical gyres: 1) wind-induced upwelling brings nutrient-rich surfaces up toward the surface, 2) mesoscale eddies transport nutrients along isopycnal surfaces into the interior of the subtropical gyre, 3) diapycnal processes and eddy-induced isopycnal heaving transfers nutrients to the euphotic zone, and 4) nutrients are remineralized and a fraction is exported to deeper layers. The thin black lines are isopycnal surfaces and the shading represents higher nutrient concentrations.

Eddy-resolving numerical simulations in idealized and realistic domains (25, 30, 31) suggest that lateral eddy transfer is an important source of nutrients to the subtropical gyres. An observational field study (11) confirmed that eddy stirring along isopycnals acts to recharge the nutrients of the gyre, particularly over the upper thermocline. Here a nutrient budget analysis in isopycnal coordinates reveals that the resupply of nutrients to the North Pacific subtropical thermocline is mediated in approximately equal contributions by particle remineralization and isopycnal eddy transport, opposing a net depletion by the mean flow below the euphotic layer. Vertical processes, including diapycnal transport and isopycnal heaving, carry these thermocline nutrients upward to the euphotic zone, where they are efficiently consumed by microorganisms and ultimately reexported. This nutrient relay (as depicted in Fig. 7) balances the loss of nutrients due to the export of organic matter through a sequence of wind-induced upwelling at the gyre flanks, along-isopycnal eddy transport into the subtropics, and upward vertical transfer to the surface.

Our analysis also reveals the vertical and horizontal structuring of nutrient transport contributions within the gyre. The effect of mesoscale eddies is visible as stirring and advection of high-nutrient waters peeling away from the flanks of the gyre and flowing along isopycnal surfaces into the bowl of the subtropical basin (Movie S1). Mesoscale eddies are preferentially generated along the flanks of the Kuroshio, North Equatorial, and North Pacific currents, sweeping nutrients both laterally toward the center of the subtropical gyre and vertically downward. The mesoscale eddy transports are most effective below the euphotic layer where they are shielded from biological depletion and penetrate further into the gyre interior. While the eddy resupply to the whole subtropical region is most important in the lower thermocline, it dominates at all levels within the recirculating gyre interior. Nutrient gradients surround the gyre margins in both the meridional and zonal directions, and zonal transport from the eastern boundary upwelling regions is significant, consistent with previous studies (43, 44). Ekman transfer of inorganic nutrients and the transport and remineralization of DOP also contribute nutrient resupply to the subtropics but most significantly close to the gyre margins and in the uppermost surface layer.

While our model study has focused on the North Pacific subtropical gyre, the inferred nutrient pathways and mesoscale eddy effects diagnosed in this work are likely relevant for the other oceanic subtropical basins. Significant contributions from mesoscale eddy stirring have been suggested by an observational field study in the North Atlantic subtropical gyre (11) and an eddy-resolving simulation spanning the North Pacific and North Atlantic basins (31). Lateral eddy transfers of nutrients along isopycnals have also been suggested as an important mechanism for the subduction of nutrients in the mode waters of the Southern Ocean and in their eventual transport to the Northern Hemisphere (45).

The salient contribution of mesoscale eddies emphasized in this work is not explicitly represented in most large-scale climate and biogeochemical models. Practical constraints also mean that the simulation examined here was eddy-permitting, leaving unresolved potentially important submesoscale dynamics, which provide enhanced vertical upwelling around frontal features and additional lateral stirring (20, 21, 23, 24, 46). While this fine-scale frontal upwelling is important in delivering nutrients from the upper thermocline to the euphotic zone, these features decay more rapidly with depth away from the surface, as compared to mesoscale eddies (47). Hence, lateral stirring by mesoscale eddies in the dark ocean is still likely to be a rate-limiting process in controlling the replenishment of nutrients in the thermocline over the entire subtropical gyre.

Climate projections suggest that surface warming will lead to an increase in upper ocean stratification, which in turn is expected to inhibit the diapycnal supply of nutrients to the euphotic zone. However, if the rate limiting process of euphotic nutrient supply is indeed set by the recharging of nutrients within the thermocline, lateral nutrient gradients and the slope of isopycnals at the gyre margins could control biological production within the gyre interior, as suggested by the isopycnal nutrient transport closure of ref. 11. In possible support of this view, some climate model projections display a more limited decline in ocean primary production when mesoscale eddies are explicitly resolved, due to a high sensitivity of the subsurface nutrient transport to model resolution (48).

In conclusion, our analysis shows that the subtropical gyre nutrient inventory is critically maintained by a three-dimensional (3D) nutrient pathway, whereby mesoscale eddies transport nutrients laterally along density surfaces, replenishing nutrients at depth within the gyre interior, which allows diapycnal mixing and vertical eddy transport to transfer nutrients up to the euphotic zone. This nutrient relay, involving both lateral and vertical transfers, provides a crucial missing step in answering the conundrum of how biological productivity is sustained over the extensive ocean subtropical gyres.

## Materials and Methods

This study makes use of the numerical simulation of ref. 49, which includes the global ocean circulation based on the Massachusetts Institute of Technology general circulation model (MITgcm) (50), coupled to the Darwin biogeochemical and ecological model (51–54). The physical model is based on the Estimating the Circulation and Climate of the Oceans configuration of the MITgcm, which constrains the simulation to observed hydrography and altimetry, through a Green’s function online adjustment of model parameters (55). The model employs a cubed-sphere grid with horizontal grid dimensions of ~18 km (56), which resolves mesoscale features in the tropics and is eddy-permitting in subpolar regions, where the radius of deformation is comparable to the grid scale. The zonal phase speed of eddies matches closely with corresponding altimeter observations (55, 57), demonstrating the model’s skill in producing realistic eddy propagation characteristics. The model also captures the broad-scale patterns of surface eddy kinetic energy inferred from altimetry but tends to underestimate its magnitude by a factor of 1.5 on average (58). The simulation is integrated from 1992 to 2003, and results are analyzed from 1994 to 2003, which allows 2 y for transient adjustments.

The marine biogeochemical model includes coupled cycles of carbon, nitrogen, phosphorus, oxygen, iron, and silica, represented in inorganic, living, dissolved, and particulate organic phases. The biogeochemical tracers interact through the formation, transformation, and remineralization of organic matter as described in ref. 51. Iron cycling includes explicit complexation with an organic ligand, scavenging by particles, and the representation of aeolian and sedimentary sources. Detritus from mortality and sinking is partitioned into dissolved and particulate pools. The dissolved organic pool is subject to 3D transport and is remineralized with a specified first-order rate constant with the same value for all elements. It is appropriate for a semilabile fraction of dissolved organic matter. Modeled particulate detritus is also subject to advection and diffusion, sinks with a constant speed, and is remineralized with a constant, first-order rate. The ecosystem component includes 51 plankton types differing in trophic strategies (autotrophs, mixotrophs, and heterotrophs), biogeochemical functions (e.g., silicifiers, calcifiers, and nitrogen fixers), and sizes (spanning from equivalent spherical diameter of 0.6 to <1,000 μm). Phytoplankton growth rates are parameterized as functions of the maximum photosynthesis rate, light, nutrient concentration, and temperature. Growth rates are determined by the most limiting resource. Algorithms and parameter values are as described in refs. 51 and 59, for plankton allometeric scaled parameters. Primary production is calculated as a function of photosynthesis rate and biomass summed across all phytoplankton types.

Here we analyze phosphate transports because the phosphorous cycle is simpler than the nitrogen cycle in the ocean, and the numerical simulation is therefore more faithful to observations. However, the strong correlation of inorganic phosphorus and nitrogen over the global ocean means that the general principles discussed here are equally significant for both elements. The nutrient budget is achieved through processing of horizontal and vertical fluxes diagnosed at 3-day output interval and recast into isopycnal coordinates through the method detailed in *SI Appendix*. Fluxes are conservatively interpolated onto a fine vertical grid of ~2 m resolution, which is employed to geometrically divide the fluxes into along- and across-isopycnal layers (60). A closed volume or mass balance can also be achieved using the same framework. Further refinements in space and time can yield more accurate results but do not affect the conclusions of this study.

While the parameterization of sinking particulate is highly idealized, the model closure maintains phosphate distributions that are close to those observed over the course of the model integration. These nutrient distributions and the observationally constrained ocean circulation together control the evaluations of phosphate transports performed in this study. Simulated primary production in the interior gyre region is slightly low compared to estimates based on remote sensing (61, 62) (*SI Appendix*, Fig. S7), in part because this configuration of the model does not account for decoupling of carbon and nitrogen stoichiometry and associated exudation (63). Nevertheless, the regional pattern of simulated primary production is plausible given the range of variability exhibited among the remote sensing–based evaluations (*SI Appendix*, Fig. S7). The export production within the gyre interior region is ~13 g C m^−2^ y^−1^ (*SI Appendix*, Fig. S3), which is consistent with the data-driven inverse model estimate of ref. 3. In common with most open ocean simulations, coastal enhancement of productivity and export in our model is weaker than in observations, although spatial patterns are otherwise comparable.

## Supplementary Material

Supplementary File

Supplementary File

## Data Availability

The setup files used to generate the numerical simulation are available at https://doi.org/10.5281/zenodo.6429906 (53), the Darwin model output is provided at https://simonscmap.com/catalog/datasets/Darwin_Nutrient (54), the satellite-based primary production estimates are available at sites.science.oregonstate.edu/ocean.productivity/index.php (62), and the isopycnal decomposition tool is posted at https://doi.org/10.5281/zenodo.6430021 (60).
