# Supplementary material for: A nutrient relay sustains subtropical ocean productivity
Source: Proc Natl Acad Sci U S A. 2022 Oct 3;119(41):e2206504119. doi: 10.1073/pnas.2206504119 (PMC9565266; doi:10.1073/pnas.2206504119)
Supplement: Supplementary File [file pnas.2206504119.sapp.pdf]

PNAS
www.pnas.org

Supplementary Information for

A nutrient relay sustains subtropical ocean productivity

Mukund Gupta, Richard G. Williams, Jonathan M. Lauderdale, Oliver Jahn, Christopher Hill, Stephanie Dutkiewicz and Michael J. Follows

Mukund Gupta.
E-mail: guptam@caltech.edu

This PDF file includes:

Supplementary text
Figs. S1 to S7 (not allowed for Brief Reports)
Legend for Movie S1
SI References

Other supplementary materials for this manuscript include the following:

Movie S1

Supporting Information Text

Nutrient budget and eddy/mean decomposition. The output provided by the numerical model allows the evaluation of a closed nutrient budget in z-coordinates, as follows:

$$\frac{\partial P}{\partial t} = -\nabla \cdot \boldsymbol{F} + B, \qquad [1]$$

where P $[mol\, m^{-3}]$ is the nutrient concentration, B $[mol\, m^{-3} s^{-1}]$ is the biological transformation term, and $\boldsymbol{F}$ $[mol\, m^{-2} s^{-1}]$ is the total nutrient flux, which includes both advective and diffusive contributions. The advective component of $\boldsymbol{F}$ represents the flux $\boldsymbol{u}P$, but is evaluated using a third-order, non-linear advection scheme with a flux limiter that ensures numerical stability. Here, $\boldsymbol{u} = (u, v, w)$ $[ms^{-1}]$ is the 3-D velocity vector in the z-coordinate system. The diffusive component of $\boldsymbol{F}$ is set to zero in the horizontal directions, and is prescribed in the vertical direction using a combination of implicit and explicit representations.

The nutrient budget of Eq. 1 is then recast into an isopycnal coordinate system using the method detailed in the section below, as follows:

$$\frac{\partial (hP_\sigma)}{\partial t} = -\nabla_\sigma \cdot (\boldsymbol{F_\sigma} h) - h\frac{\partial F_d}{\partial \hat{d}} + hB_\sigma, \qquad [2]$$

where h $[m]$ is the isopycnal layer thickness. P_σ $[mol\, m^{-3}]$ and B_σ $[mol\, m^{-3} s^{-1}]$ are the nutrient concentration and the biological transformation terms evaluated in isopycnal coordinates, respectively. $\boldsymbol{F_\sigma}$ $[mol\, m^{-2} s^{-1}]$ is the sum of nutrient fluxes directed along isopycnals, including advective and diffusive contributions, with zonal and meridional components F_σ^x and F_σ^y, respectively. The advective component of $\boldsymbol{F_\sigma}$ effectively represents the product of isopycnal velocity and nutrient concentrations $\boldsymbol{u_\sigma} P_\sigma$, where $\boldsymbol{u_\sigma} = (u_\sigma, v_\sigma)$ $[ms^{-1}]$ is the velocity along isopycnals. F_d $[mol\, m^{-2} s^{-1}]$ is the diapycnal nutrient flux, directed across isopycnals and including advective and diffusive contributions. The advective component of F_d effectively represents $u_d P_\sigma$, where u_d $[ms^{-1}]$ is the velocity across isopycnals.

The relative contributions of the eddy and mean components of the flow are evaluated via a Reynolds decomposition of the time-averaged nutrient budget in isopycnal coordinates given by:

$$\frac{\partial (\overline{hP_\sigma})}{\partial t} = -\nabla_\sigma \cdot (\overline{\boldsymbol{F_\sigma} h}) - \overline{h\frac{\partial F_d}{\partial \hat{d}}} + \overline{hB_\sigma}. \qquad [3]$$

The Reynolds decomposition is carried out for the isopycnal convergence and biological source terms, and ignored for the tendency and diapycnal convergence terms as their fluctuating components are expected to be negligible. The time-mean and layer-integrated isopycnal flux $\overline{\boldsymbol{F_\sigma} h}$ is composed of an Eulerian mean component $(\boldsymbol{F_\sigma} h)^{mean}$ and an eddying component $(\boldsymbol{F_\sigma} h)^{eddy}$, such that:

$$\overline{\boldsymbol{F_\sigma} h} = (\boldsymbol{F_\sigma} h)^{mean} + (\boldsymbol{F_\sigma} h)^{eddy}, \qquad [4]$$

where the Eulerian mean component is defined as:

$$(\boldsymbol{F_\sigma} h)^{mean} \equiv \bar{\boldsymbol{u_\sigma}} \bar{P_\sigma} \bar{h} \qquad [5]$$

and the eddying component is obtained as a residual from $\overline{\boldsymbol{F_\sigma} h}$. $\boldsymbol{u_\sigma}$ is the flow velocity along isopycnal surfaces, and the overbar represents a time averaging over the simulation period. $(\boldsymbol{F_\sigma} h)^{eddy}$ includes the combined effects of the fluctuating along-isopycnal velocity $\boldsymbol{u'_\sigma}$, isopycnal layer thickness h' and tracer concentration P'_σ, due to the transient mesoscale eddies resolved by the simulation. $(\boldsymbol{F_\sigma} h)^{eddy}$ also includes the diffusive component of the flux along isopycnals, but this contribution is expected to be minor relative to the resolved eddying advective flux.

Performing the Reynolds decomposion and substituting Eq. 4 and 5 into Eq. 3 gives:

$$\underbrace{\frac{\partial (\overline{hP_\sigma})}{\partial t}}_{\text{Tendency}} + \underbrace{\nabla_\sigma \cdot (\bar{\boldsymbol{u_\sigma}} \bar{P_\sigma} \bar{h})}_{\substack{\text{Iso div} \\ \text{mean}}} + \underbrace{\nabla_\sigma \cdot (\boldsymbol{F_\sigma} h)^{eddy}}_{\substack{\text{Iso div} \\ \text{eddy}}} + \underbrace{\overline{h\frac{\partial F_d}{\partial \hat{d}}}}_{\text{Dia div}} = \underbrace{\bar{h}\bar{B_\sigma}}_{\text{Mean bio}} + \underbrace{\overline{h' B'_\sigma}}_{\text{Eddy bio}} \qquad [6]$$

The terms in Eq. 6 represent, in order: (a) the total tendency of nutrient concentrations, (b) the convergence of the Eulerian mean isopycnal nutrient flux, (c) the divergence of the eddying isopycnal nutrient flux, (d) the divergence of the diapycnal nutrient flux, (e) the mean component of biological source term, and (f) the eddying component of the biological term. A similar layer-averaged nutrient budget is evaluated in (1) for the subtropical North Atlantic, exploring in situ observations to infer diapycnal mixing and eddy stirring. This layer-averaged tracer budget for a density layer is also disscussed in (2, 3).

Integrating Eq. 6 zonally from the western coast x_w to the eastern coast x_e, and meridionally between latitudes y_1 and y_2, gives:

$$
\begin{aligned}
&\int_{y_1}^{y_2}\int_{x_w}^{x_e} \frac{\partial(\overline{hP_\sigma})}{\partial t}\,dx\,dy \;+\; \int_{x_w}^{x_e}\left[\bar{v_\sigma}\bar{P_\sigma}\bar{h} + \overline{(F_\sigma^y h)}^{eddy}\right]_{y_2} dx \;-\; \int_{x_w}^{x_e}\left[\bar{v_\sigma}\bar{P_\sigma}\bar{h} + \overline{(F_\sigma^y h)}^{eddy}\right]_{y_1} dx \;+\; \int_{y_1}^{y_2}\int_{x_w}^{x_e} \overline{h\frac{\partial F_d}{\partial \hat{d}}}\,dx\,dy \\
&= \int_{y_1}^{y_2}\int_{x_w}^{x_e} (\bar{h}\bar{B}_\sigma + \overline{h'B'_\sigma})\,dx\,dy
\end{aligned}
\tag{7}
$$

The zonal fluxes in the mean and eddy isopycnal divergence terms of Eq. 6 disappear when integrating over the basin, and only the meridional components remain, zonally-integrated and evaluated at latitudes y_1 and y_2 (terms 2 and 3 in Eq. 7). Eq. 7 is used to calculate the meridional fluxes, tendencies and convergences presented in Fig. 4 of the paper.

Isopycnal decomposition. Results from this study rely on performing a nutrient budget in an isopycnal coordinate system, in order to separate adiabatic processes from diabatic ones. Since the MITgcm model is configured in a z-coordinate system, an offline post-processing algorithm is designed to achieve this decomposition in a conservative manner, as illustrated in Fig. S1 and detailed below:

1. The original vertical grid is refined into sub-cells with a customizable resolution. This resolution may differ for each of the original (irregular) vertical grid lines, such that these remain a subset of the refined grid. This avoids complications associated with splitting the horizontal fluxes in a conservative manner and is more computationally efficient. All operations are conducted on area-integrated fluxes. Vertical fluxes (defined at the top and bottom of each cell) and quantities defined at the center of the cells (e.g. density and nutrient concentration) are linearly interpolated in the vertical direction. Horizontal fluxes (defined at the zonal and meridional edges of the grid) are split equally within each sub-grid cells.

2. Sub-cells belonging to a given isopycnal range are identified by selecting the vertical sub-cells most closely bounding the σ_0 range at each horizontal grid point. Finer resolution of the refined grid allows for a more accurate selection. Note that this step relies on the water column being stably stratified (σ increasing uniformly with depth).

3. At each horizontal grid point, the fluxes bounding the relevant sub-cells selected in the previous step are divided into isopycnal and diapycnal quantities. Along isopycnal fluxes are defined as exchanges with neighboring cells belonging to the isopycnal sub-range, whereas diapycnal fluxes are defined as exchanges with neighboring cells that do not belong to that sub-range.

4. The area-integrated fluxes are summed separately for isopycnal fluxes entering the sub-range from the 'left', isopycnal fluxes exiting the domain from the 'right', diapycnal fluxes entering the domain from the bottom and diapycnal fluxes exiting the domain from the top. Here, 'left/right' refer to the 'west/east' and the 'south/north' edges of the cells for the zonal and meridional directions, respectively.

For this study, the isopycnal decomposition is performed with a vertical grid size of approximately 2 m, and a time resolution of 3 days. Further refinements in the vertical do not affect the results, and fluxes do not display significant variability beyond the weekly timescale. When an isopycnal layer outcrops, the algorithm is not carried out and that time step is ignored in the time-averaged flux. Coastal boundaries are handled with the same partial cell method as in the original grid. The resulting nutrient budget has machine-precision residual values.

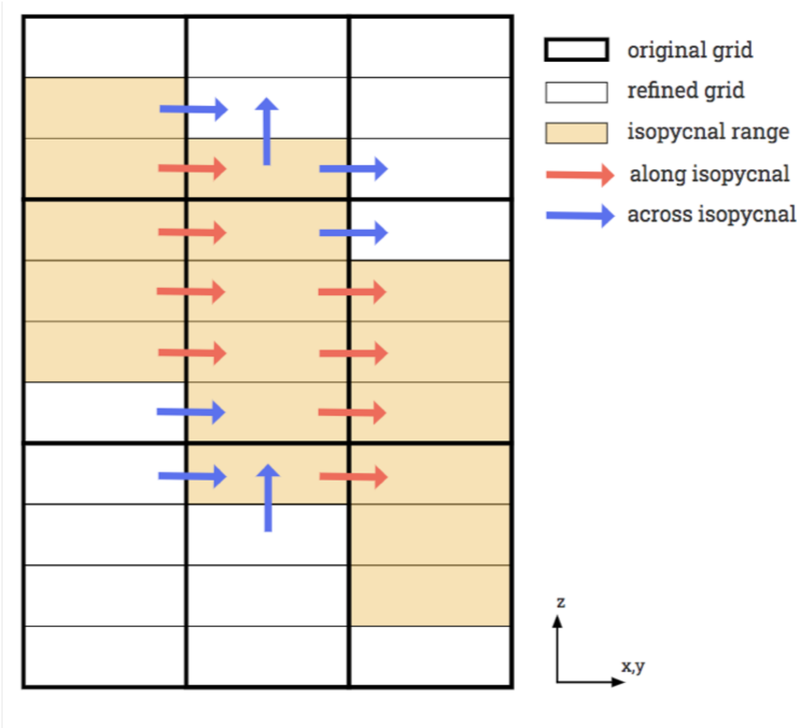

Fig. S1. Schematic representation of the procedure employed to decompose horizontal and vertical fluxes into fluxes along and across isopycnal layers. The bold lines depict a section of the original model grid over which the simulation is integrated (not to scale). The thinner black lines represent the vertically refined grid used for post-processing, and the shaded cells represent the isopycnal range of interest evaluated over the refined grid. Orange arrows depict exchanges between the shaded cells (along the isopycnal layer), whereas blue arrows are exchanges between the isopycnal layer and the neighbouring exterior cells (across the isopycnal layer). At each horizontal grid point, this decomposition yields zonal and meridional fluxes along the isopycnal, as well as diapycnal fluxes from the top and bottom of the layer. This decomposition is exact for the mass or volume fluxes, as well as the tracer fluxes.

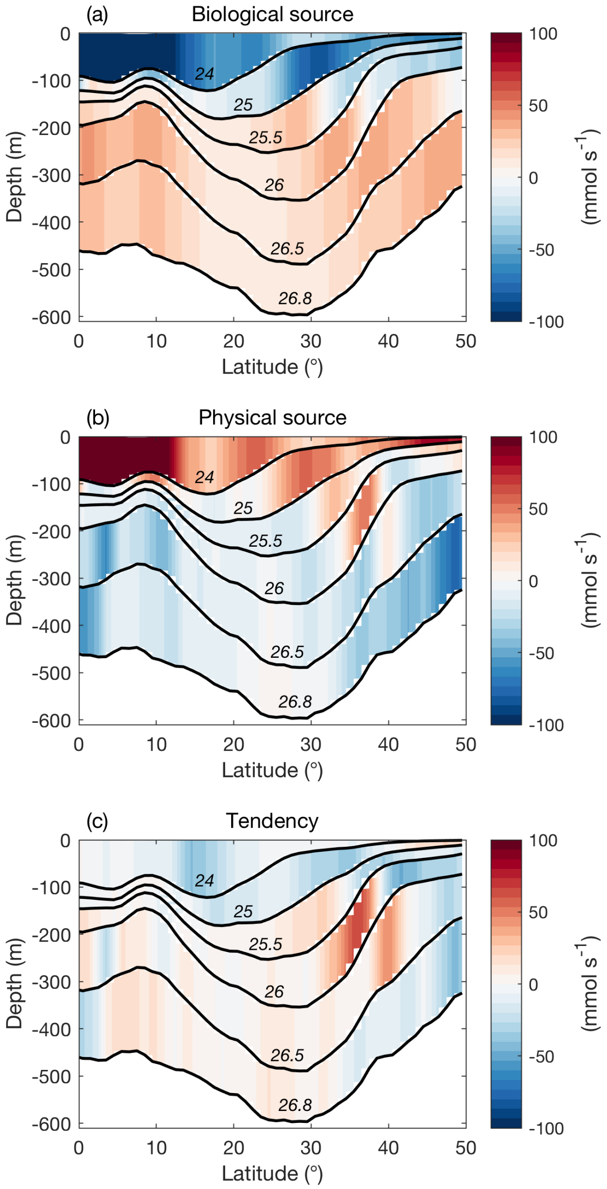

Fig. S2. Volume-integrated phosphate budget performed in isopycnal coordinates according to Eq. 2 and plotted in depth space. (a) Biological source term, (b) physical source term equal to the sum of isopycnal and diapycnal convergences, and (c) tendency term.

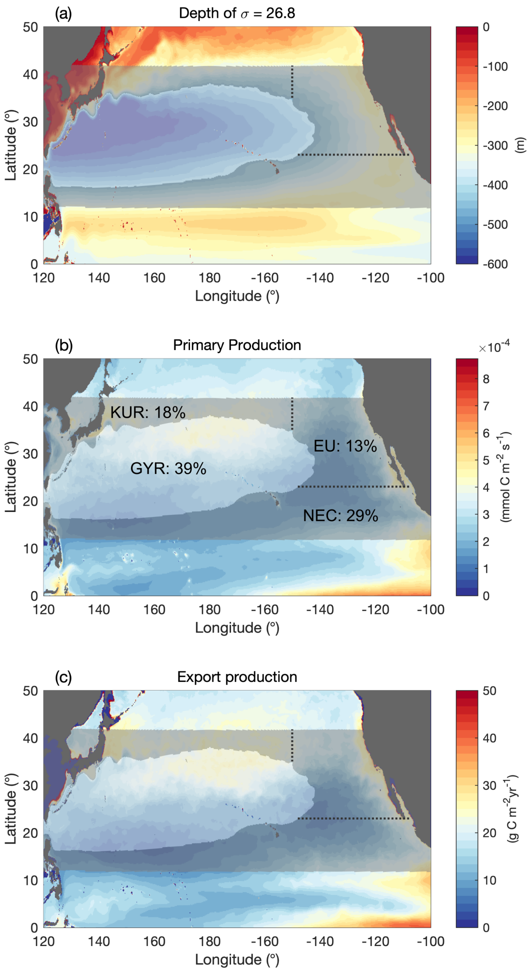

Fig. S3. (a) Depth of the σ = 26.8 isopycnal, (b) depth-integrated primary production, and (c) export production at 150 m depth. The subtropical basin is defined as the region between 12°N and 42°N. The recirculating gyre interior (GYR) is defined as a subset of the subtropical basin, where the depth shown in panel (a) is larger than $d_g = 450$ m. The darkly shaded regions indicates the rest of the subtropical basin, subdivided into the North Equatorial Current (NEC), Kuroshio (KUR) and Eastern Upwelling (EU) sections, according to a constant longitude line at -150°E (dotted vertical line) and a constant latitude line at 23°N (dotted horizontal line). The percentages shown in panel (b) indicate the proportion of primary production occurring within each sub-region.

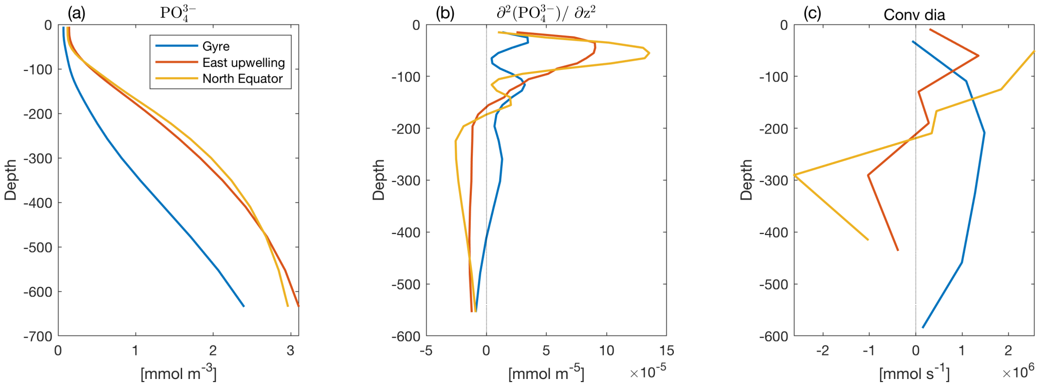

Fig. S4. Horizontally-averaged profiles for the sub-regions shown in Fig. S3, namely the gyre interior, North Equatorial Current and Eastern Upwelling sections. The Kuroshio region is omitted, due to strong variabilty over the decadal period. (a) Phosphate concentration, (b) second derivative of the phosphate concentration profile, and (c) diapycnal flux convergence. The quantities in panels (a) and (b) are in depth coordinates, whereas those in panel (c) are in isopycnal coordinates plotted in depth space. The sign of the second derivative of phosphate concentrations tends to match with the sign of the diapycnal flux convergences, as one would expect from a one dimensional diffusive process. We infer that the deep nutricline within the gyre interior, driven by large-scale Ekman downwelling, leads to positive diapycnal convergence throughout the top 600 m of the water column. On the other hand, the shallower nutricline in upwelling regions leads to a diapycnal source in the upper 200 m, and diapycnal depletion between 200 - 600 m.

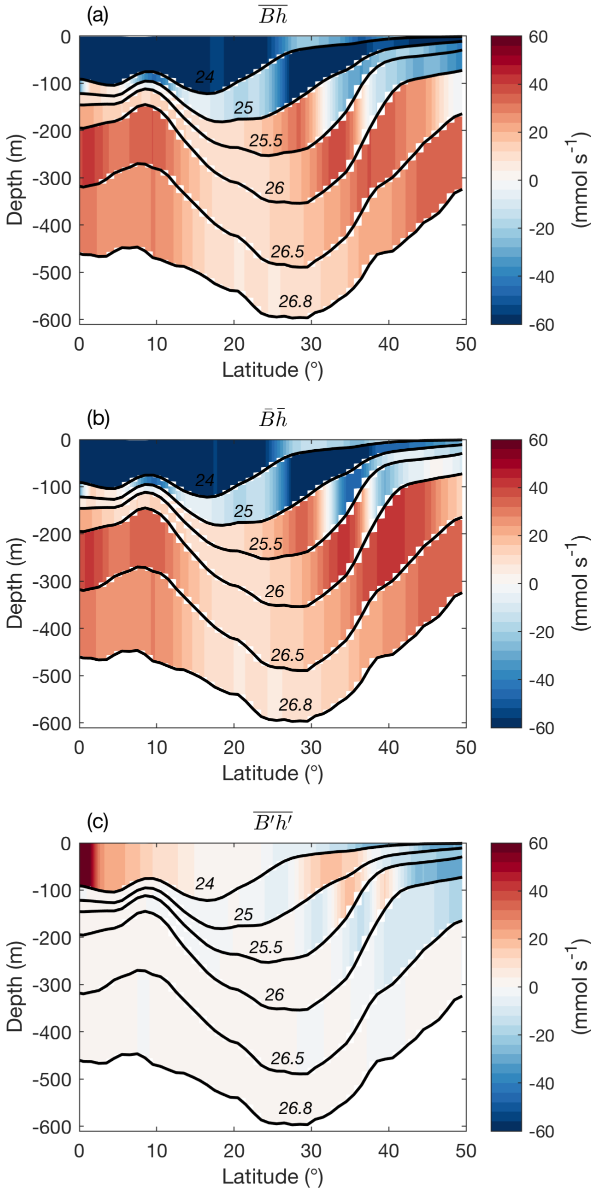

Fig. S5. Zonally-averaged and thickness-weighted biological source term evaluated in isopycnal coordinates and plotted in depth space. (a) Net source term, (b) contribution from the time-mean biological source, and (c) fluctuating component involving time-correlations between the layer thickness and the biological source.

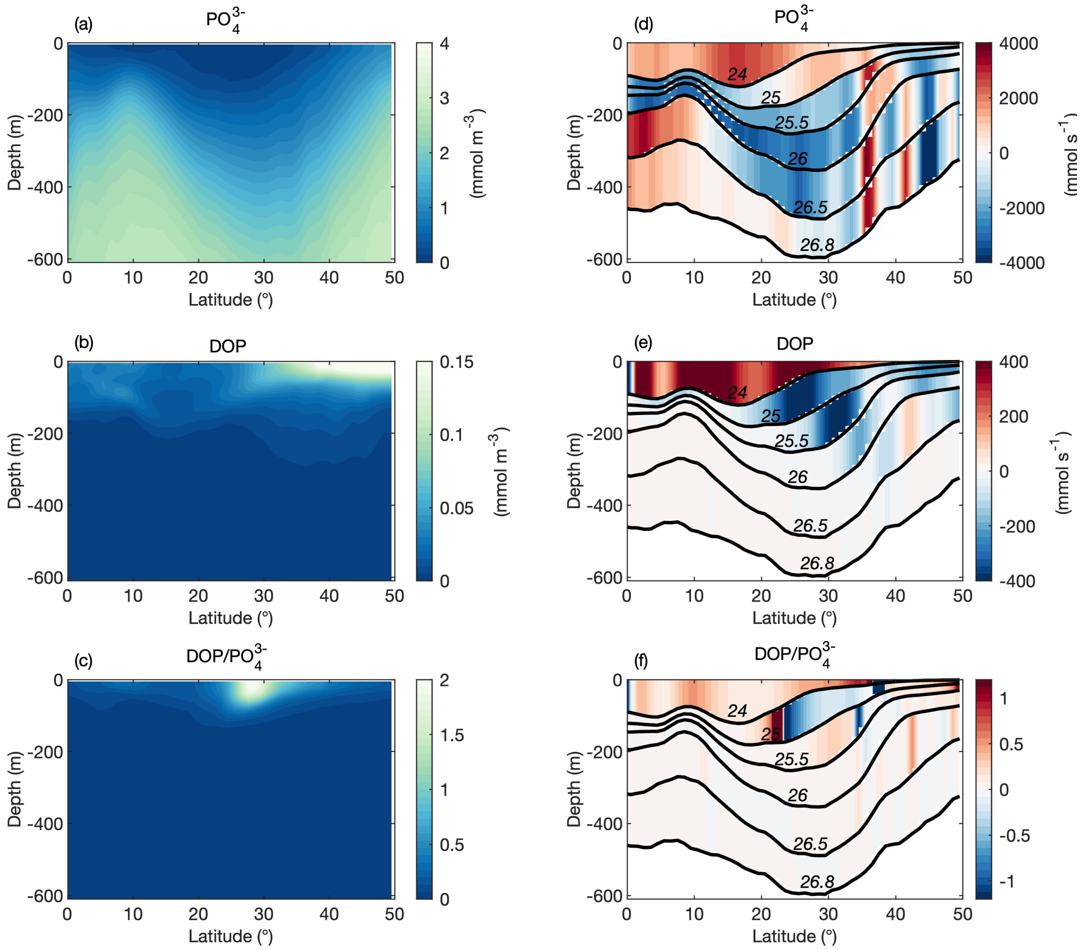

Fig. S6. Zonally-averaged concentrations (left) and meridional fluxes (right) of PO_4^{3-} (top) and DOP (middle). The bottom panels show the corresponding ratios of $\mathrm{DOP}/\mathrm{PO}_4^{3-}$ for concentration (c) and meridional flux (f).

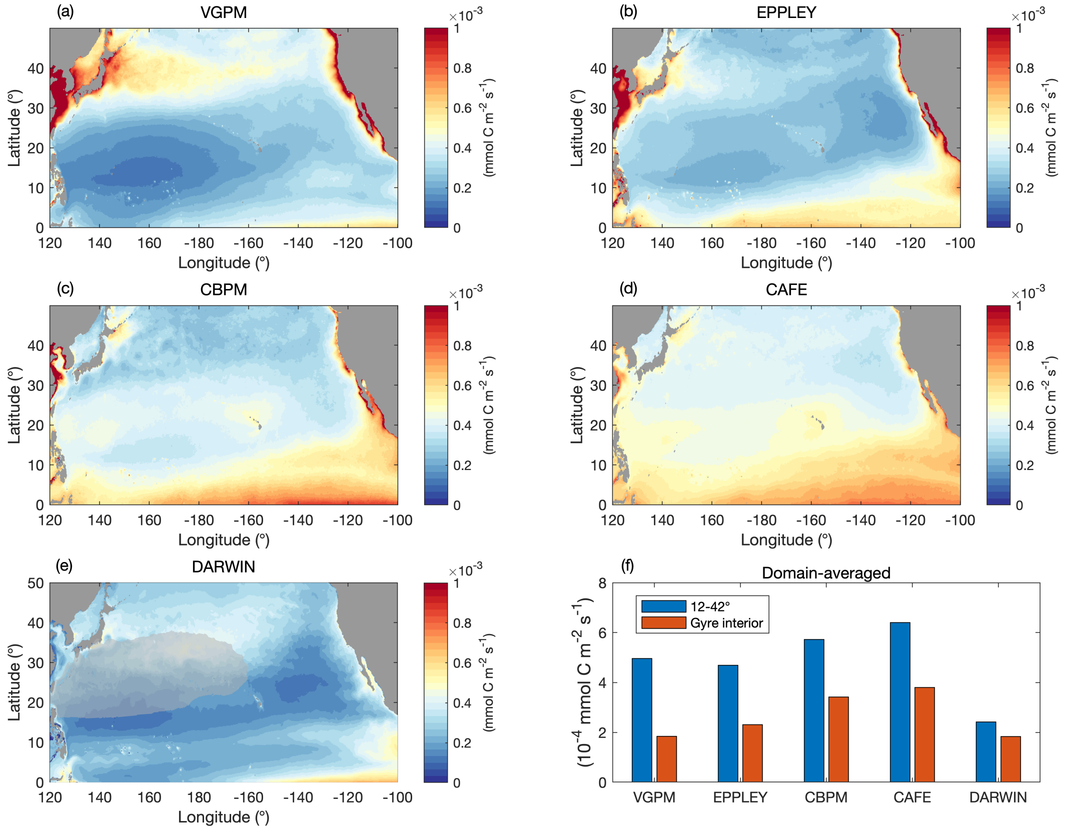

Fig. S7. Primary production obtained from the SeaWifs satellite ocean color inferences (4), using the (a) VGPM, (b) EPPLEY, (c) CPBM and (d) CAFE algorithms, averaged between 1998-2003. (e) Corresponding primary production in the Darwin model. (f) Primary production averaged between 12°- 42° latitudes (blue), and the gyre interior (orange) shown as the grey shading in panel (e).

Movie S1. **PO_4^{3-} concentration along the $\sigma_0 = 26.0$ isopycnal over the North Pacific subtropical basin (filled contours) and depth of the isopycnal surface with 10 m contour intervals (line contours). Land is depicted in black and isopycnal outcrops are shown in dark grey.**

References

1. CP Spingys, et al., Observations of nutrient supply by mesoscale eddy stirring and small-scale turbulence in the oligotrophic north atlantic. *Glob. Biogeochem. Cycles* **35**, e2021GB007200 (2021).
2. R Bleck, *Ocean Modeling in Isopycnic Coordinates*, eds. EP Chassignet, J Verron. (Springer Netherlands, Dordrecht), pp. 423–448 (1998).
3. TJ McDougall, The relative roles of diapycnal and isopycnal mixing on subsurface water mass conversion. *J. Phys. Oceanogr.* **14**, 1577 – 1589 (1984).
4. MJ Behrenfeld, PG Falkowski, Photosynthetic rates derived from satellite-based chlorophyll concentration. *Limnol. Oceanogr.* **42**, 1–20 (1997).
